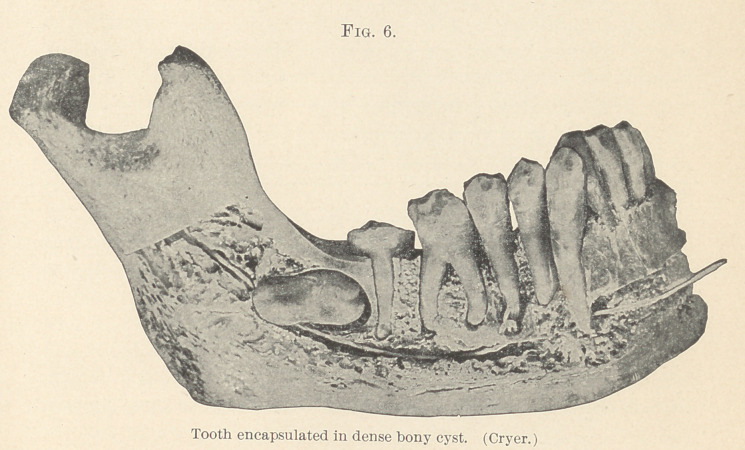# Surgical Aspects of Disturbed Dentition of the Third Molars2Read at Portland, Ore., July 12, 1905, before the Section on Stomatology of the American Medical Association. Published with consent of the Journal of the American Medical Association.

**Published:** 1905-11

**Authors:** M. L. Rhein

**Affiliations:** New York City


### International Dental Journal.

Vol. XXVI. November, 1905. No. 11.

### Original Communications.[1]

### SURGICAL ASPECTS OF DISTURBED DENTITION OF THE THIRD MOLARS.[2]

BY M. L. RHEIN, M.D., D.D.S., NEW YORK CITY.

The third molars generally make their appearance between the ages of sixteen and twenty. The final development of the length of the maxillæ posterior to the mental foramen in the lower jaw and the maxillary sinus in the upper, takes place during this period, and in this manner sufficient space is provided for the proper alignment of the third molar when it breaks through the gum tissue. Where this development proceeds without any disturbing constitutional causes, no difficulties attend the eruption of these teeth. As, however, all inflammatory actions that arise during this period, either from local pathological causes or by virtue of constitutional disturbances, have a marked effect on the osseous development of these parts, we find the normal type of development to be the exception. These departures from a purely normal type vary greatly according to the amount of disturbance that has taken place,

[1] The editor and publishers are not responsible for the views of authors of papers published in this department, nor for any claim to novelty, or otherwise, that may be made by them. No papers will be received for this department that have appeared in any other journal published in the country.

[2] Read at Portland, Ore., July 12, 1905, before the Section on Stomatology of the American Medical Association. Published with consent of the Journal of the American Medical Association.

and in a large number of cases interfere with the normal eruption of the third molars. This interference with the eruption of these teeth is confined more particularly to the lower jaw. The reason for the lower third molar being exceptionally liable to serious disturbances is due to the difference in the anatomy of the two jaws. On account of the limitations of time, and small percentage of troubles encountered with erupting upper molars, these remarks will be confined strictly to the inferior teeth.

The necessity for sufficient room at the angle of the body and the ramus is shown in Fig. 1 (Cryer). Fig. 1 shows the mandible of one of the Fan tribe of West Africa, and there is abundance of room for the third molar. Fig. 2 (Cryer) is a picture of a Caucasian lower jaw of normal type where there is just barely sufficient room for the third molar. The lessening of this amount of space is one of the most prolific sources of disturbed dentition in this locality. Too frequently there is no room left between the second molar and the ramus, and as a result the third molar finally erupts in some unusual position. During this effort at unnatural eruption, grave disturbances are very liable to occur. The close proximity of the cribriform tube (Fig. 3, Cryer) to the roots of the third molar is another anatomical feature which has a marked bearing on the etiology of disturbances in this region. In many anatomical specimens the incompleted roots can be seen penetrating the canal itself. The cribriform tube or inferior dental canal (Fig. 4, Cryer) furnishes a pathway of slight resistance to infection, and once involved leads to serious septic conditions. Another marked pathological condition, that results from the close proximity to the inferior dental nerve, is that the irritation of this nerve is very likely to produce a stimulation in nutritional supply at this point, so that the normal cancellated bone (Fig. 5, Cryer), through which the erupting tooth can readily force its way, is replaced by the hardest of osseous structure which attempts to force the tooth aside and seek some easy but unnatural mode of eruption. This excitation of bony development sometimes proceeds to such extremes as to encapsulate the tooth in a dense bony cyst (Fig. 6, Cryer), in which it becomes impacted. The study of this condition will not be considered at this time, but it is merely mentioned as a possible result where this undue eruption proceeds to its extreme limit.

The unnatural increase in osteoblasts with all its attending inflammatory conditions, produces a marked effect on all the sur-

Fig. 1.

Mandible of a member of the Fan tribe, West Africa, showing abundance of room for third molar. (Cryer.)

Fig. 2.

Caucasian lower jaw of modern type, with barely sufficient room for third molar. (Cryer.)

Fig. 3.

Close proximity of cribriform tube to root of third molar. (Cryer.)

Fig. 4.

Showing cribriform tube. (Cryer.)

Fig. 5.

Normal cancellated bone. (Cryer.)

Fig. 6.

Tooth encapsulated in dense bony cyst. (Cryer.)

rounding tissue. Their tone of resistance is depressed in proportion to the degree of irritation, and this is also further influenced by any disturbances of the general system. When this decreased resistance has reached a certain stage, the parts become an easy prey to infection. In this way we find the eruption of the third molar, which should be a natural physiological action, complicated by more or less severe pathological disturbances.

Another etiological factor, that sometimes plays an important rôle in depressing the local vitality, is found in mouths where on occlusion the two jaws come so close together that traumata of the gums over the erupting tooth are constantly taking place, caused by the upper teeth constantly biting the gum tissue over the erupting tooth, and infection frequently ensues.

Another marked point of irritation is found in such mouths where, on account of lack of space between the second molar and the ramus, the third molar is pushed forward and its eruption is impeded by coming in contact with the distal side of the second molar. Frequently only the slightest impingement on the territory of the second molar will start up an inflammatory action, and the parts become rapidly infected.

Since 1828, when Toirac gave the first accurate description of this disturbance, French and German authorities have written extensively on this subject. In 1878 Heydenreich reviewed the list of writers on this subject up to that date. According to Magitot and David, complications ensue in the eruption of seventy-five per cent. of third lower molars. Some very unique etiological theories are advanced by some of these authorities. Moty, in 1901, tries to show an analogy between suppurating dermoid cysts and infections of erupting third molars. He finds the cause at the end of the root, and speaks of it as an "epithelial inclusion," and it will be instructive to quote from him. He says, "In our opinion these phlegmons are due entirely to a collection of epithelial cells enclosed at the bottom of the alveolus. This enclosed epithelium is found as a fungous mass which has gradually infiltrated the wall of the alveolus without enlarging the latter. In some cases this neoplastic tissue becomes encysted. In nearly all cases these epithelial inclusions cause abscesses at the time of eruption of the wisdom-teeth, or later on. Early extraction may be necessary on account of the pain, even before suppuration has set in, but the latter invariably occurs in cases left to themselves. The cause of this non-bacterial suppuration is due entirely to these cells acting as a foreign body.

"Epithelial inclusions may be found with other teeth, but only rarely. It is very probable that the enamel body may leave an epithelial focus in the gums which may develop into a tooth (giving rise to a third dentition). These cases themselves are very rare, but grouped with other anomalies (single cusps, an additional cusp or round root added to a molar, a large tooth, an extra tooth not growing from the gums, etc.) make a large class. Abscesses associated with healthy wisdom-teeth and commencing in the depth of the alveolus are generally sterile. The pus has no bad odor unless a secondary infection has supervened. An odor generally indicates a carious tooth."

If views such as these are correct, it upsets all our ideas of the pathogenic conditions at work in these cases. It is wise, however, to have our attention directed to the different views that are held on this subject, and I have quoted the latter one because it is so remarkably well expressed.

Careful observation of clinical data impress us with the view that there are many predisposing factors to be considered in the etiological study of different cases.

For the sake of convenience the subject can be considered in two divisions. First, disturbances which antedate the appearance of the tooth. Second, those which take place after the partial eruption of the tooth. Symptoms in the early stages are not much more marked than in ordinary physiological dentition. There is the usual amount of pain accompanied by congestion of the gums overlying the erupting tooth. The œdema becomes more marked, rapidly progressing in every direction, involving the pillars of the fauces and the various glands in the mouth. Neuralgic pains radiate to the ear and eye, and as the submaxillary glands become involved, they extend to the neck, shoulders, and even to the arms. The patient finds it more and more difficult to open the mouth. As infection progresses, the pulse becomes more rapid, and temperature may even rise to 103° F. A careful digital examination over the gums will readily detect the presence of the tooth underneath. In some cases the gum becomes attenuated and pale, as in ordinary dentition, but, as a rule, this is not the case, but the opposite condition results, which is that of excessive congestion.

Prompt excision of the entire hood of gum tissue is at once called for. The resection of the gum should be so thorough as to completely expose the four sides of the tooth. Many authorities recommend the cautery for this purpose, but the knife is a more valuable adjunct, as the blood-letting itself is very beneficial. Every possible means should now be used to aid in the rapid eruption of the tooth and the prevention of reinfection of the parts. Foreign writers all unite in a plea for extraction as the only radical cure in these cases. Whenever it becomes evident that the third molar, by reason of its irregular position and a lack of space in the jaw for its proper berth, can never become a useful organ, the earliest extraction of the tooth is called for.

On the other hand, in a large percentage of cases, the tooth can be brought into proper alignment and occlusion, and under such circumstances there is no valid excuse for its extraction. A strip of gauze should be packed between the gum and tooth around its entire circumference. In case of the second molar acting as an obstruction, this gauze will act to some extent as a wedge in making a proper place for the third molar. The gauze packing should be frequently changed. If possible, the mouth should be sprayed at intervals with a hot borinated wash, which, at any rate, should be used by the patient frequently as a mouth lotion. The focus of infection in most cases under sufficiently energetic treatment will soon find an outlet, either into the mouth or the fauces, and the symptoms will rapidly abate.

The trismus, paralysis, and œdema symptoms in these cases involve so much surrounding territory that they are frequently mistaken by the medical attendant for adenitis, stomatitis, pyorrhœa, parotitis, and diseases due to other teeth. An error in diagnosis in these cases generally means an error in treatment, and usually results in an unnecessary disfigurement of the face. The frequency of errors in diagnosis of these cases is one of the strongest pleas that can be made for the education of medical men in the principles of dentistry.

The following notes taken from a typical case will illustrate this fact:

Miss A., between the ages of sixteen and seventeen, had been convalescing for two weeks from an attack of measles, when she commenced to have paroxysms of pain in the posterior portion of the mouth. This was accompanied by œdema and a steadily rising temperature. The physician who had attended her with the measles was sent for, and he made a diagnosis of adenitis of the submaxillary glands. The temperature twenty-four hours later had risen to 102.5° F., and her pulse was 120. Under his direction, Credé's ointment was liberally spread over the neck at the angle of the jaw, and this was covered by rubber tissue. The expectation of the attending physician was that the absorption of the silver salt would arrest the infective infiltration. The result, however, was that it acted as a poultice, and drew the inflammation and infection within the mouth to the outer tissues.

Forty-eight hours after this treatment had been commenced I was called to see the patient, because the mother had a suspicion that the teeth might be involved, the patient having continuously complained of pain at the angle of the body and the ramus of the mandible.

*Examination.*—I found the patient suffering severely from neuralgic pains, with a great amount of œdema, extending through the entire cervical region, and involving most of the hyoid muscles. A very careful external examination failed to discover any sign of any glandular enlargement, but simply an œdema penetrating through all the tissues. On this account it was difficult for the patient to open her mouth, yet a careful digital examination over the mandible, between the second molar and the ramus, readily distinguished the outlines of an erupting third molar. The attending physician had reached the point where he expected to call in a surgeon and make an external incision into the infected area.

*Treatment.*—The case being turned over to me, I at once dissected away the entire hood of gum tissue which was covering the erupting tooth, and found that the mesial approximal surface of the third molar was impinging slightly on the distal approximal contour of the second molar. Iodoform gauze was packed around the entire circumference of the tooth, special attention being paid to the space between the second and third molars.

*Result.*—The temperature of the patient dropped immediately to 100.5°, and the pulse to 100. Naturally, the ointment and rubber tissue were at once removed from the neck, and hot borinated mouth lavations were ordered every fifteen minutes, with the hope of bringing the inflammatory action back again into the oral cavity. A blood-count showed twenty thousand leukocytes.

There were many unpleasant instances connected with this case on acount of the ill-feeling engendered in the mind of the physician, because of the necessity of my making so radical a change in the treatment of the patient.

The patient was constantly under the care of a trained nurse, and for the following five days the temperature varied between 99.8° and 101°. At this time results of the treatment prevailed, and a purulent effusion made its escape from the tonsils. The packing of the gauze between the two molars was persisted in for about ten days, when sufficient space between the two molars was obtained, and the third molar was finally erupted into a position of correct occlusion and alignment.

The most serious type of cases are those where disturbances do not abate after partial eruption of the tooth has taken place. Writers frequently speak of the purulent troubles of third molars that exist even after eruption of the tooth has been completed, but a careful anatomical examination will generally show that the eruption of the tooth has not been entirely completed, some obstacle being present which prevents the tooth coming out as far as it naturally would. This is the class of cases that Moty, quoted above, speaks of, in which he classifies the cause as an "epithelial inclusion."

The undue osteoblastic stimulation that would result from the inflammatory action present in these cases is sufficient to account for this condition, which he so graphically describes as "epithelial inclusion," but which is nothing more than an overstimulation of peridental membrane, which, if persisted in, frequently leads to a complete ossification of the parts and produces an impacted tooth.

In all these cases every effort should be made in the line of proper orthodontia, so that, if possible, the tooth can be properly erupted and preserved. The value of the retention of the third molar in a mandible large enough to contain it should never be overlooked. If, however, it is found impossible to bring the tooth to its proper height and alignment, extraction should be resorted to at the very earliest moment. In such cases it makes no difference how difficult it is to extract the tooth; if necessary deep narcosis must be resorted to, and a portion of the mandible cut away, so as to remove every portion of the tooth. The longer there is any delay in such cases, the greater is the danger of severe neuralgic complications and infiltrative osteomyelitis through the passageway of the inferior dental canal. For this same reason, if extraction of such a tooth has taken place, the greatest care should be taken to keep the wound packed with sterile gauze, in order to avoid reinfection which may lead, by means of the cribriform tube, to an extensive osteomyelitis. The same rule obtains in cases of abscessed or necrotic conditions where there is any danger of reinfection, and especially where the field of operation is in close proximity to any of the osseous sinuses. This does not necessarily include healthy alveolar sockets in other parts of the jaws.

The following clinical case will illustrate the danger resulting from neglecting to attend to the sterilization of such a socket:

Mr. S., bachelor, aged about thirty years, had a third molar extracted which had never completely erupted, but had been a constant source of irritation for many years. The extraction was performed by a specialist in this line, and was said to have been a very difficult one. The later surgical operation demonstrated definitely that the roots of the third molar penetrated the cribriform tube, as shown in Fig. 3 (Cryer). Inadequate attention to keeping the wound sterile (no packing having been used) was followed by an infection in the alveolar sockets. This spread without any difficulty to the inferior dental canal, and when I saw the patient for the first time in the hospital on July 3, 1900, in consultation with Dr. Howard Lilienthal, he was not far from a moribund state. There was a very rapid and weak pulse, with a temperature of 105.5°. Our diagnosis was an infiltrative osteomyelitis progressing through the passageway of the cribriform tube. The patient was immediately anæsthetized, and an external incision made at the angle of the body and the ramus. The bone was chiselled away at this point until the interior of the tube was exposed, where there was an effusion of a large mass of purulent matter, grayish in color, and most foul in odor.

The condition of the patient at this time was so serious that the operation was made as short and rapid as possible, great fear being entertained that he would not survive the ordinary surgical shock. Drainage was established through the external opening thus obtained, and the recovery was very slow. It soon became evident that an entire infected zone of bone had not been removed, and on October 27 a subperiosteal resection was performed, extending from the symphysis of the body to a considerable portion of the ascending ramus. After this the case went on to complete recovery.

Figs. 4 and 5, taken from Cryer, will illustrate most beautifully the parts operated upon. The prognosis in such cases is remarkably good, as long as correct surgical principles are used in the operative procedure.

Summarizing, it would appear that the medical profession are remiss in their failure to call on stomatologists for consultation in obscure cases of infection in the oral regions. Frequent errors of diagnosis made by medical men have been the cause of numerous cases of unnecessary facial disfigurement.

On the other hand, the stomatologists themselves should realize the value of retaining all of the molars, if possible. When extraction has to be resorted to, too much and too harsh criticism cannot be used against those members of this specialty who are negligent in taking proper precautions against infection of wounds in this locality.

### BIBLIOGRAPHY.

1. Jourdain: Revue Médicale, 1829, p. 396.
2. Thirly: Geneva, 1857.
3. Robertson: Dental Cosmos, vol. v., 1863, p. 243.
4. Heydenreich: 1878.
5. Magitot: 1879.
6. Trudeau: Medical Examiner, vol. i., 1838–58.
7. Cobleigh: Cin. Med. News, 1880, vol. ix. p. 433.
8. Magitot: Contribution à l'étude des accidents de l'éruption de la dent de sagesse inférieure, Gaz. hebd. de méd., vol. xvi. p. 3.
9. Delacour: Accidents de l'éruption de la dent de sagesse, Bull. Soc. Anat. Clin., 1892, vol. vii. p. 169.
10. Gutman: Verhandlung d. deutsch. odont. Gesellschaft, 1892, vol. iii. p. 292.
11. Dunogier: Contribution à l'étude des accidents lies à l'évolution de dents de sagesse, Mém. et Bull. Soc. Méd. et Chirurg., 1893–94, p. 11.
12. Davezac: Gaz. des hôp. de Toulouse, 1894, p. 25.
13. Demons: Gaz. des hôp. de Toulouse, 1894.
14. Redier: Des accidents de l'éruption de la dent de sagesse, Journ. de Soc. Médicale de Lille, 1895.
15. Bourgogne: Un cas de lymphangite, Odontologie, 1897, v. p. 254.
16. Heydenreich: Rev. Méd. de l'est, Nancy, 1898, vol. xxx. p. 285.
17. CauMartin: Accidents provoqués par l'éruption de la dent de sagesse, L'Écho Méd. du Nord, Lille, 1901, v. p. 15.
18. Moty: Accidents de la dent de sagesse, Rev. de Chirurg., vol. xxiii., 1901, p. 617.
19. Beltrami: Sur quelques dus à l'évolution de la dent de sagesse, Marseilles, Méd., 1902, vol. xxxix. p. 429.

20. Nogue: Arch. de Stomatol., 1902, vol. iii. p. 590.
21. Witzel: D. Monatschrift f. Zahnheilkunde, 1902, vol. xx. p. 590.
22. Guittermin, L.: Schweizerische Vierteljahrschrift für Zahnheilkunde, January, 1903.
23. Williger: D. Monatschrift f. Zahnheilkunde, 1903, vol. xxi. p. 57.
24. Kollbrunner: Schweizerische Vierteljahrschrift für Zahnheilkunde.
25. Turnbul: Dental Record, 1903, vol. xxi. p. 36.
26. Frey: L'Odontologie, October 30, 1904.
27. Dunogier: Gaz. des hôpitaux de Toulouse, 1894, vol. vii. p. 17.
28. Cornudet: De la dent de sagesse, Lille, 1887.
29. Chevassu: De quelques accidents causés par l'éruption et les déviations de la dent de sagesse, Paris, 1873.
30. dit Orme: De quelques accidents causés par l'éruption de la dent de sagesse, Montpelier, 1880.
31. Gallas: Quelques considerations sur les accidents dus à l'éruption de la dent sagesse inférieure, Bordeaux, 1889.

## Figures and Tables

**Fig. 1. f1:**
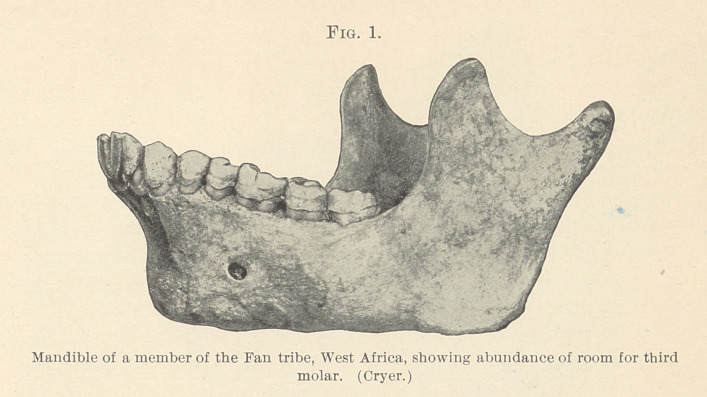


**Fig. 2. f2:**
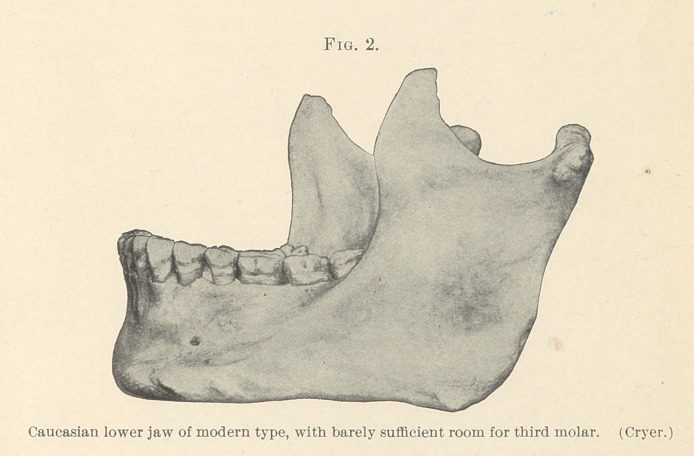


**Fig. 3. f3:**
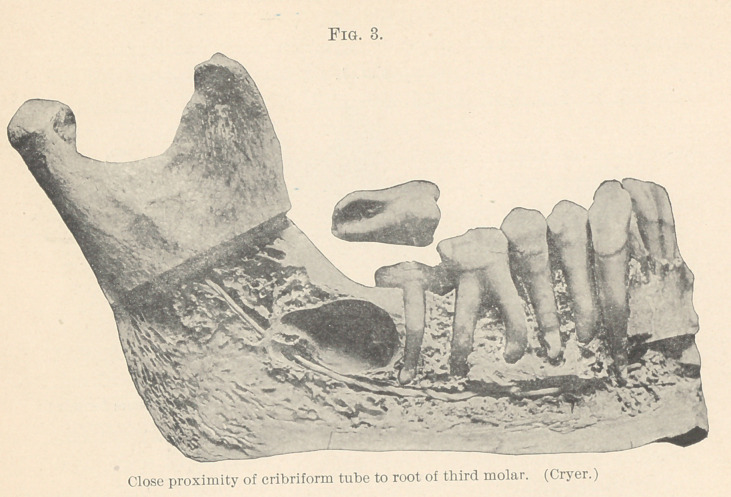


**Fig. 4. f4:**
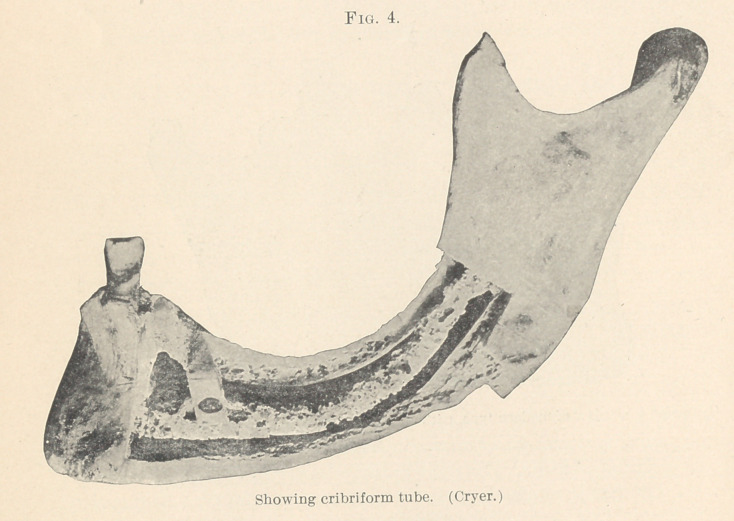


**Fig. 5. f5:**
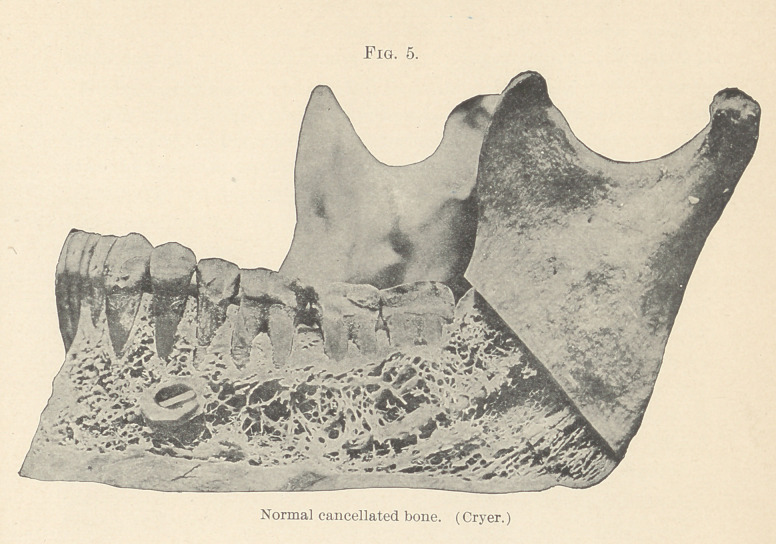


**Fig. 6. f6:**